# Aquaporin-8 promotes human dermal fibroblasts to counteract hydrogen peroxide-induced oxidative damage: A novel target for management of skin aging

**DOI:** 10.1515/biol-2022-0828

**Published:** 2024-02-16

**Authors:** Shu-Hsiang Liu, Wei-Chun Lin, En-Chih Liao, Yung-Feng Lin, Ching-Shuen Wang, Sheng-Yang Lee, Dee Pei, Chun-Hsien Hsu

**Affiliations:** School of Nursing, College of Nursing, National Taipei University of Nursing and Health Sciences, Taipei, Taiwan; School of Dental Technology, College of Oral Medicine, Taipei Medical University, Taipei, Taiwan; Department of Medicine, MacKay Medical College, New Taipei, Taiwan; Institute of Biomedical Sciences, MacKay Medical College, New Taipei, Taiwan; School of Medical Laboratory Science and Biotechnology, College of Medical Science and Technology, Taipei Medical University, Taipei, Taiwan; School of Dentistry, College of Oral Medicine, Taipei Medical University, Taipei, Taiwan; Department of Dentistry, Wan-Fang Medical Center, Taipei Medical University, Taipei, Taiwan; Department of Internal Medicine, Division of Endocrinology and Metabolism, Fu Jen Catholic University Hospital, New Taipei, Taiwan; Department of Family Medicine, Taipei City Hospital, Heping Fuyou Branch, No. 12, Fuzhou St., Zhongzheng Dist., Taipei City 100, Taiwan (R.O.C.); Wanhua District Health Center, Department of Health, Taipei City Government, Taipei, Taiwan; School of Medicine, College of Medicine, Fu Jen Catholic University, New Taipei, Taiwan; Department of Exercise and Health Sciences, University of Taipei, Taipei, Taiwan; Department of Family Medicine, Cardinal Tien Hospital, New Taipei, Taiwan; Department of Family Medicine, Shin Kong Wu Ho-Su Memorial Hospital, Taipei, Taiwan

**Keywords:** aquaporin-8, dermal fibroblasts, hydrogen peroxide, oxidative stress, aging

## Abstract

The skin is subjected to various external factors that contribute to aging including oxidative stress from hydrogen peroxide (H_2_O_2_). This study investigated the distribution of aquaporin-8 (AQP8), a protein that transports H_2_O_2_ across biological membranes, in skin cells, and its effects in mitigating H_2_O_2_-induced oxidative damage. Human dermal fibroblasts were treated with increasing concentrations of H_2_O_2_ to evaluate oxidative damage. Cell viability, reactive oxygen species (ROS) generation, and the expression of specific genes associated with skin aging (IL-10, FPR2, COL1A1, KRT19, and Aggrecan) were evaluated and AQP8 expression was assessed via quantitative polymerase chain reaction and western blotting. Small-interfering RNA was used to silence the AQP8 gene and evaluate its significance. The results show that H_2_O_2_ treatment reduces cell viability and increases ROS generation, leading to oxidative damage that affects the expression of target molecules. Interestingly, H_2_O_2_-treated cells exhibit high levels of AQP8 expression and gene silencing of AQP8 reverses high levels of ROS and low levels of COL1A1, KRT19, and Aggrecan expression in stressed cells, indicating that AQP8 plays a vital role in preventing oxidative damage and consequent aging. In conclusion, AQP8 is upregulated in human dermal fibroblasts during H_2_O_2_-induced oxidative stress and may help prevent oxidative damage and aging. These findings suggest that AQP8 could be a potential therapeutic target for skin aging. Further research is necessary to explore the feasibility of using AQP8 as a preventive or therapeutic strategy for maintaining skin health.

## 1 Introduction

The skin, being the largest organ in the human body, serves as a protective barrier against environmental factors. Nevertheless, it is exposed to various stressors that lead to the production and accumulation of reactive oxygen species (ROS), resulting in oxidative stress, which is considered a crucial contributor to skin aging. One of the hallmarks of skin aging is the degradation of extracellular matrix (ECM) proteins, including elastin, collagen, and proteoglycans [1,2]. Dermal fibroblasts are the primary cell type in the dermis responsible for producing most ECM components [3]. Studies have indicated that oxidative stress in dermal fibroblasts is associated with the progression of skin aging [4–6]. Thus, developing strategies to boost the ability of dermal fibroblasts to counteract ROS-induced oxidative stress could be a promising approach to managing skin aging.

Aquaporins (AQPs) are small membrane proteins that facilitate the transport of water and small molecules across biological membranes. Thirteen members of the AQP family (AQP0-12) are expressed throughout the body [7]. Animal studies have identified the expression of several AQPs, including AQP1, AQP3, AQP4, AQP7, and AQP9, in skin tissues, with lower expression levels observed in aged mice compared to young mice [8]. Moreover, recent experimental findings have revealed that AQP5 expression, which is essential for the balance between epidermal stem cell proliferation and differentiation, is reduced with age [9]. The decreased expression of AQPs in skin cells and tissues during aging suggests that these channels may play a vital role in skin physiology, making them promising targets for cosmetics and pharmaceuticals aimed at preventing skin aging and enhancing skin resistance.

Recently AQPs initially described as bidirectional water transporters [10,11] have been found to transport H_2_O_2_ [12]. Specifically, aquaporin-8 (AQP8) is a transmembrane protein that transports water and H_2_O_2_, expressed in the mitochondrial and plasma membranes of different cell types, including human colonic epithelial cells, mouse 3T3-L1 preadipocytes, and rat insulin-producing cells [13–15]. Furthermore, studies have suggested that AQP8 features highly efficient H_2_O_2_ transport functions of various rat AQP family members overexpressed in HEK-293 cells [16–18]. It is worth noting that not only AQP8 but also AQP11 engage in concerted subcellular coordination to facilitate the transport of H_2_O_2_, as documented in the previous studies [19,20]. As such, our first decision to exclude AQP11 from our consideration came from the data deposited in the Human Protein ATLAS database, which indicates predominant AQP8 protein expression in skin fibroblasts, whereas AQP11 demonstrates broader expression across various cell types identified in skin. Therefore, we hypothesized that could play an important role in regulating ROS transportation in skin. H_2_O_2_ is a ROS that can lead to oxidative damage to biomolecules and contribute to skin aging when combined with free transition metal ions, or when present alone [21,22]. Therefore, H_2_O_2_ exposure is a commonly used oxidant model in studies of oxidative stress and to study the role of AQP8 in skin cells.

This study aimed to determine AQP8 expression in human dermal fibroblasts and test the hypothesis that AQP8 promotes the ability of these cells to counteract H_2_O_2_-induced oxidative damage. AQP8 has a pronounced rescuing effect against H_2_O_2_-induced oxidative damage, which could protect human dermal fibroblasts from aging. This finding suggests that AQP8 may play a crucial role in the protection of skin cells from oxidative stress and contribute to maintaining skin health and youthfulness. This discovery of AQP8’s protective effect on human dermal fibroblasts is significant since it may pave the way for developing novel strategies to prevent or reduce skin aging by targeting AQP8 activity. Further research is needed to elucidate the precise mechanisms by which AQP8 exerts its protective effect and its potential role as a target for skincare products and pharmaceuticals.

## 2 Materials and methods

### 2.1 Cell culture

The human skin fibroblast cell line (CG1639) was purchased from the Bioresource Collection and Research Center (BCRC, Hsinchu, Taiwan) and cultured in 85% Dulbecco’s modified Eagle’s medium (DMEM, ThermoFisher Scientific, Waltham, USA) with 4 mM l-glutamine supplemented with 4.5 g/L glucose, 1.5 g/L sodium bicarbonate, and 15% fetal bovine serum at 37°C in a humidified atmosphere of 5% CO_2_.

### 2.2 Establishment of H_2_O_2_-induced skin damage model

A previous study reported a model in which stimulation of dermal fibroblasts with H_2_O_2_ was associated with skin aging and increased the expression of inflammatory molecules [23]. In this study, H_2_O_2_ was also used to establish the skin damage model using the CG1639 cell line. The cells were seeded in six-well tissue culture plates and exposed to increasing concentrations of H_2_O_2_ (100, 200, 400, 800, and 1,200 μM) for the indicated times.

### 2.3 Cell viability assay

Cell viability was determined by 3-(4,5-dimethylthiazol-2-yl)-2,5-diphenyl tetrazolium bromide (MTT, Sigma Aldrich, St. Louis, USA) assay following the manufacturer’s instructions. The absorbance was measured at 570 nm using a spectrophotometer and cell morphology was observed under the light microscope (Olympus, Shinjuku, Japan).

### 2.4 Cell oxidative assay

Cell oxidative assay was performed using CellROX™ Green Reagent (ThermoFisher Scientific, Waltham, USA). The cells were seeded at a density of 3 × 10^5^ cells per well in 6-well tissue culture plates for 24 h. After treatment with increasing concentrations of H_2_O_2_ for 3 h, CellROX^®^ Reagent at a final concentration of 5 μM was added to the cells and incubated for 30 min. Subsequently, the cells were washed three times with phosphate-buffered saline and fixed with 4% formaldehyde for 15 min for examination using a fluorescence microscope.

### 2.5 Quantitative polymerase chain reaction (qPCR)

Total RNA was isolated using RNAzol^®RT^ and reversely transcribed according to the manufacturer’s instructions before qPCR using the PowerUp SYBR Green Master Mix (ThermoFisher Scientific, Waltham, USA). The primer sequences were as follows (IDT, California, USA): AQP8, forward 5′-GCGAGTGTCCTGGTACGAAC-3′ and reverse 5′-CAGGCACCCGATGAAGATGAA-3′; IL-10, forward 5′-TACCACCTCCCGAAAATGTCA-3′ and reverse 5′-CCCAGTCTGAATGCTCATCTG-3′; FPR2, AGTCTGCTGGCTACACTGTTC-3′ and reverse, 5′-TGGTAATGTGGCCGTGAAAGA-3′; COL1A1, forward 5′-GAGGGCCAAGACGAAGACATC-3′ and reverse 5′-CAGATCACGTCATCGCACAAC-3′; KRT19, forward 5′-ACCAAGTTTGAGACGGAACAG-3′ and reverse 5′-CCCTCAGCGTACTGATTTCCT-3′; Aggrecan, forward 5′-ACTCTGGGTTTTCGTGACTCT-3′ and reverse 5′-ACACTCAGCGAGTTGTCATGG-3′; GAPDH, forward 5′-TGTGGGCATCAATGGATTTGG-3′ and reverse 5′-ACACCATGTATTCCGGGTCAAT-3′. The expression of target genes was normalized to the internal control (GAPDH), and relative gene expression levels were presented.

### 2.6 Western blotting

The cells were harvested and lysed on ice using the cell lysis buffer (Cell Signaling, Danvers, USA). The supernatant was collected by centrifugation at 12,000 *g* for 15 min at 4°C and the protein concentration was determined using the bicinchoninic acid protein detection kit. Equal amounts of protein (40 μg) were loaded and separated on 10% sodium dodecyl sulfate polyacrylamide gel electrophoresis gels and subsequently transferred onto polyvinylidene difluoride membranes (GE Healthcare, Chicago, USA). The membranes were blocked with a blocking buffer for 2 h at room temperature before incubation with the following primary antibodies, anti-AQP8 (Invitrogen, Waltham, USA, PA5-103616, 1:1,000), anti-Cola1 (GeneTex, Irvine, USA, GTX112731, 1:1,000), anti-KRT19 (Invitrogen, Waltham, USA, MA5-15884, 1:1,000), or anti-β-actin (GeneTex, Irvine, USA, GTX11003, 1:1,000) at 4°C overnight. The membranes were incubated with horse-radish peroxidase-linked secondary antibodies and the immunoreactive bands were detected by a professional imaging system.

### 2.7 Silencing of AQP8 expression using small-interfering RNA (siRNA)

To knock down AQP8, the cells were cultured for 24 h in a medium containing 10% FBS, transfection reagent, AQP8 siRNA, and nontargeted siRNA (10 nM each). Then, the cells were cultured in serum-free medium in the absence (6 and 24 h) and presence of high (+100 mM) NaCl (6 h) and CoCl_2_ (150 µM; 24 h), respectively, or in a 0.2% O_2_ atmosphere (24 h).

### 2.8 Statistical analysis

Three independent experiments with cell lines from different donors were performed for each test. Data are shown as mean ± standard error of the mean. A post hoc test was employed following analysis of variance to assess differences among the groups using Prism (GraphPad Software, San Diego, USA). A *p*-value of less than 0.05 was considered statistically significant.

## 3 Results

### 3.1 H_2_O_2_ induces oxidative damage in human dermal fibroblasts

**Figure 1:**
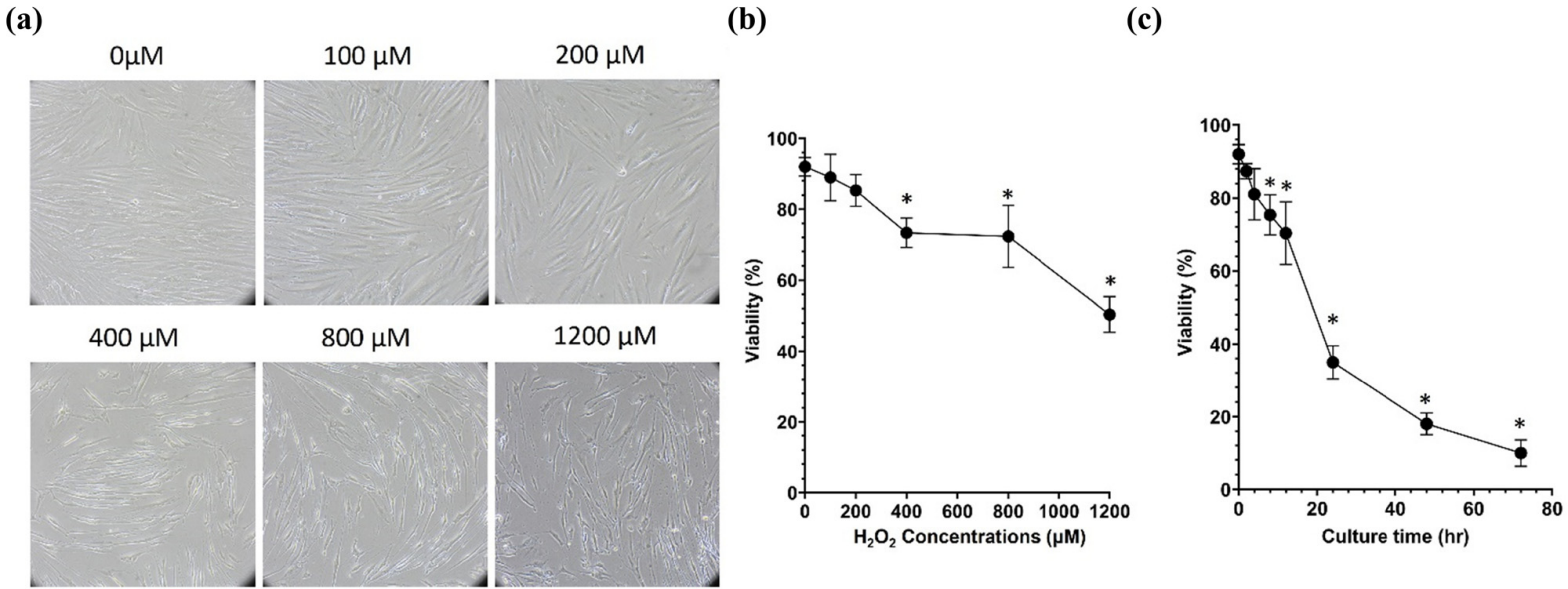
H_2_O_2_ treatment induces cytotoxicity in human dermal fibroblasts. (a and b) The cells were exposed to increasing concentrations of H_2_O_2_ (100, 200, 400, 800, and 1,200 μM) for 24 h. (a) The cell morphology was observed under a light microscopy (10×). (b) The cell viability was determined using an MTT assay. (c) The cells were exposed to H_2_O_2_ (800 μ M) for different time intervals. The cell viability was determined using an MTT assay. *p  <  0.05, as compared to the control. All experiments were performed independently at least three times.

**Figure 2:**
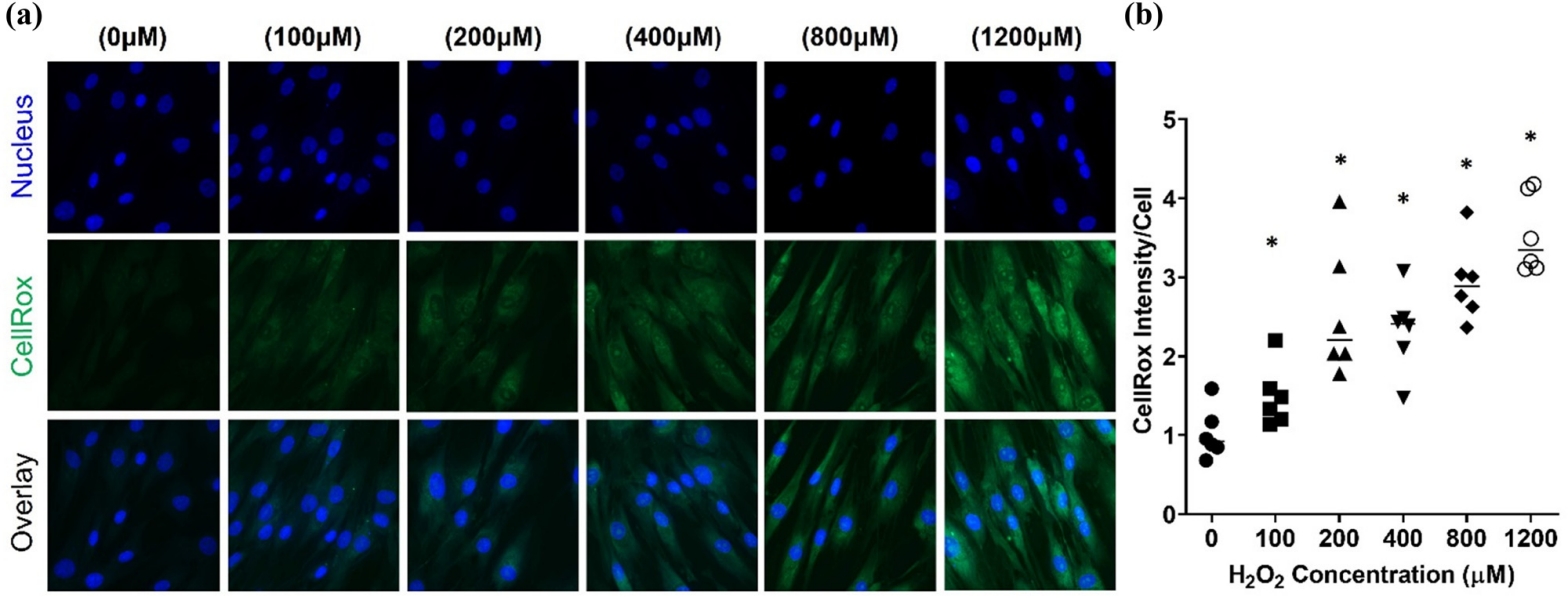
H_2_O_2_ treatment induces oxidative stress in human dermal fibroblasts. The cells were exposed to increasing concentrations of H_2_O_2_ (100, 200, 400, 800, and 1,200 μM) for 3 h. Intracellular ROS generation was detected using CellROX™ Green Reagent. (a) Representative fluorescent microscopy images of ROS detection. Green foci represent CellROX dye upon oxidation by ROS and binding to DNA (blue). (b) Quantitative analysis of (a). *p  <  0.05, as compared to the control. All experiments were performed independently at least three times.

**Figure 3:**
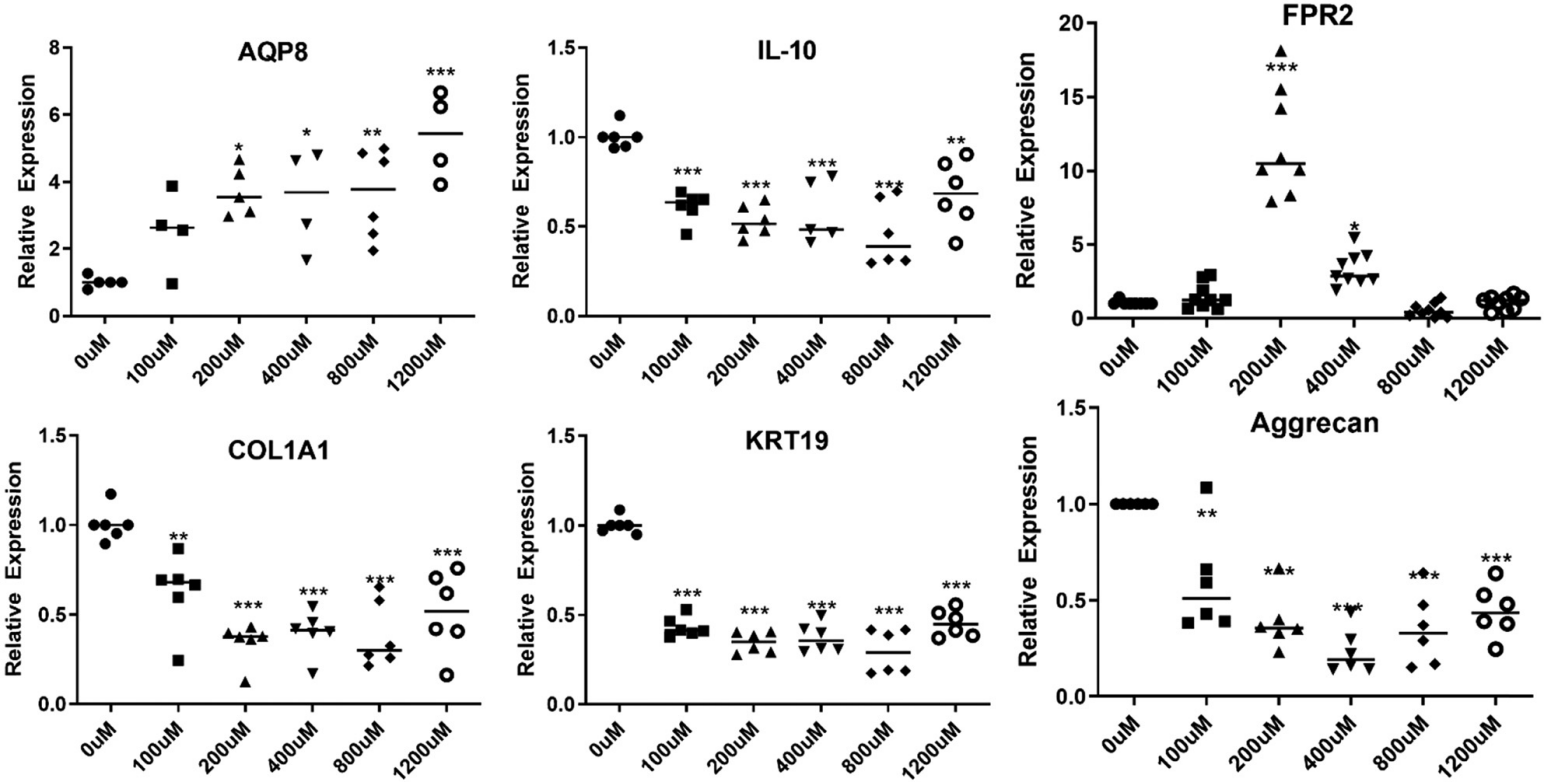
Analysis of gene expression in human dermal fibroblasts. The cells were exposed to increasing concentrations of H_2_O_2_ (100, 200, 400, 800, and 1,200 μM) for 3 h. The expression level of target gene was identified by qPCR. *p  <  0.05, **p  <  0.01, ***p  <  0.001, as compared to the control. All experiments were performed independently at least three times. Abbreviations: AQP8, aquaporin-8; IL-10, interleukin-10; FPR2, *N*-formyl peptide receptor 2; COL1A1, collagen type I1; KRT19, keratin 19.

**Figure 4:**
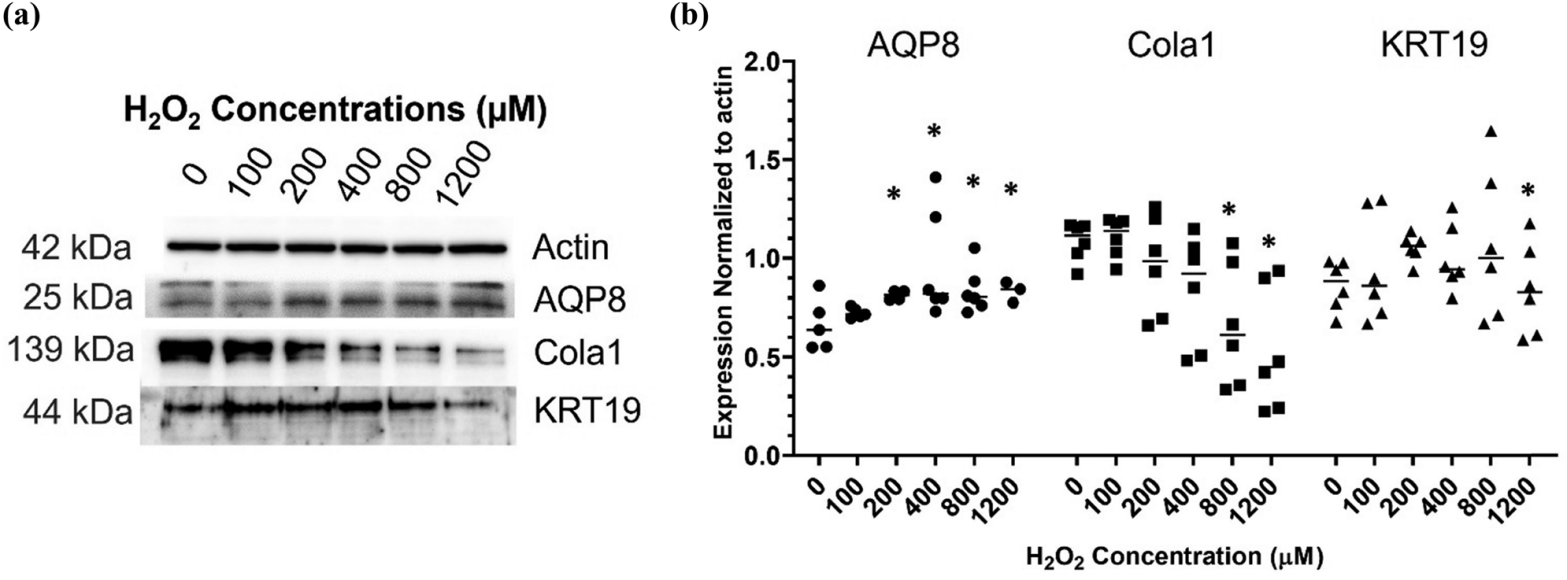
Analysis of protein expression in human dermal fibroblasts. The cells were exposed to increasing concentrations of H_2_O_2_ (100, 200, 400, 800, and 1,200 μM) for 3 h. The protein expression of target molecule was determined by Western blot analysis. (a) Representative immunoblotting images of protein expression. (b) Quantitative analysis of (a). *p  <  0.05, as compared to the control. All experiments were performed independently at least three times. Abbreviations: AQP8, aquaporin-8; Cola1, collagen type I α1; KRT19, keratin 19.

To determine the deleterious effects of ROS-induced oxidative stress in human dermal fibroblasts, the human skin fibroblast cell line CG1639 was stimulated with increasing concentrations (100, 200, 400, 800, and 1,200 μM) of H_2_O_2._ The cytotoxic effects of H_2_O_2_ in these human dermal fibroblasts were determined after 24 h of treatment and as shown in Figure 1a, H_2_O_2_-treated cells showed lower cell density and they were morphologically less elongated, wider, and less fusiform compared to control cells. The higher H_2_O_2_ the concentrations, the more evident the changes. Consistently, the MTT assay indicated that the H_2_O_2_ treatment dose-dependently reduced the cell viability (Figure 1b). Additionally, H_2_O_2_ significantly decreased cell viability in a time-dependent manner (Figure 1c). Next, the ability of H_2_O_2_ to induce oxidative damage in the human dermal fibroblasts CG1639 was determined after 3 h of treatment. As shown in Figure 2, H_2_O_2_ treatment dose-dependently enhanced the levels of ROS, indicative of oxidative stress in the exposed cells. In addition, qPCR analysis (Figure 3) showed that the expression of anti-inflammatory cytokine IL-10 was significantly decreased in H_2_O_2_-treated cells compared to control cells. Meanwhile, a significant increase in the gene expression of *N*-formyl peptide receptor 2 (FPR2), which is involved in the pathogenesis of chronic inflammatory diseases [24], was observed after treatment with H_2_O_2_ at 200 μM (*p* < 0.001) and 400 μM (*p* < 0.05). Furthermore, our data indicated that the gene expression of COL1A1 (encoding collagen type I1 [Cola1]), KRT19 (encoding keratin type I cytoskeletal 19 [KRT19]), and Aggrecan (encoding aggrecan proteoglycan), which are important for the structural integrity of ECM, were dose-dependently downregulated by H_2_O_2_ treatment. Consistently, western blot analysis showed a dose-dependent decrease in the protein expression of Cola1 and KRT19 in H_2_O_2_-treated cells compared to control cells (Figure 4). Taken together, our results suggest that stimulation of human dermal fibroblasts with H_2_O_2_ is associated with skin aging by decreasing cell viability, increasing oxidative stress, dysregulating the expression of inflammatory molecules, and suppressing the expression of ECM and cytoskeleton components.

### 3.2 AQP8 promotes human dermal fibroblasts to counteract H_2_O_2_-induced oxidative damage

**Figure 5:**
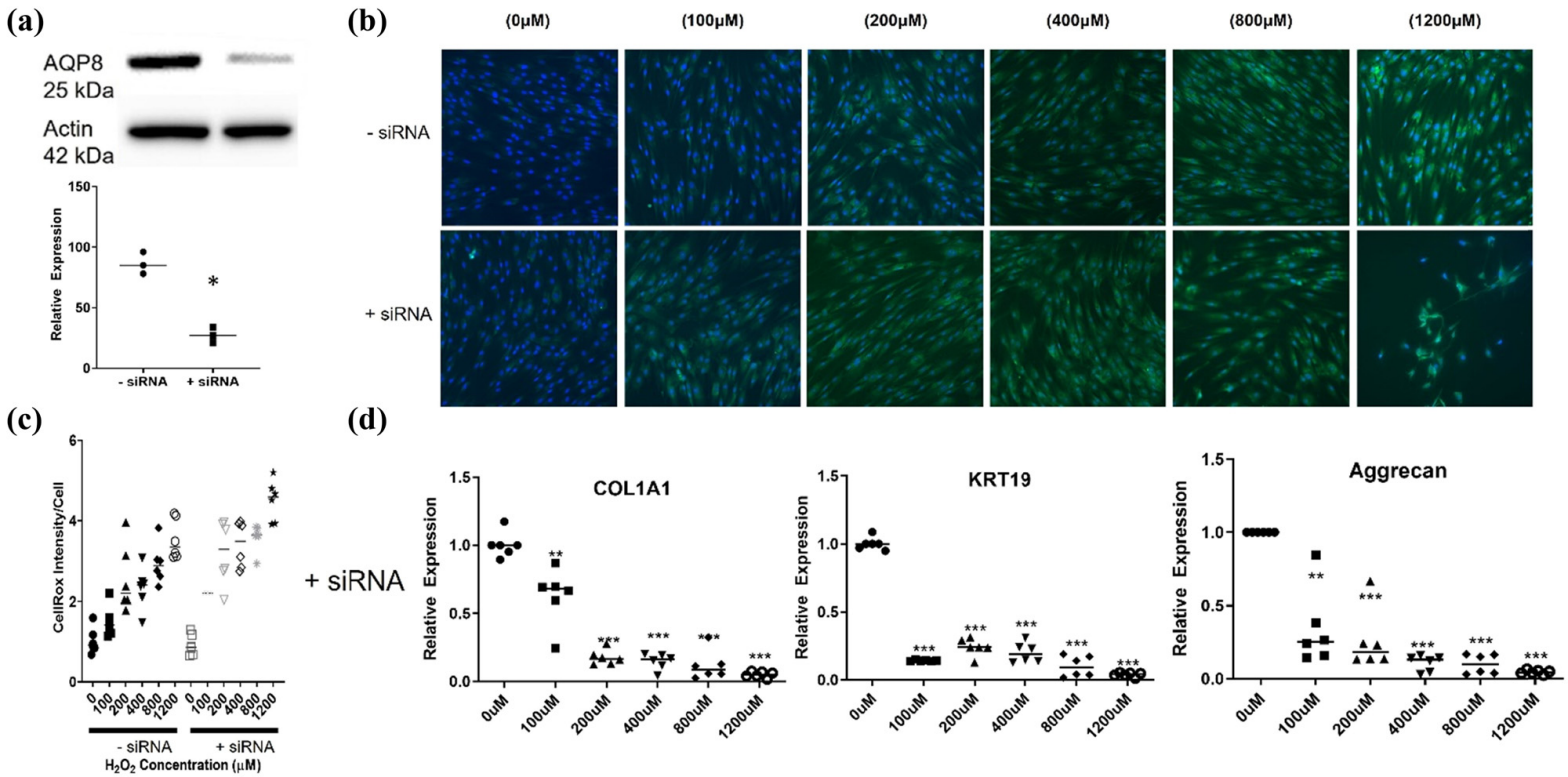
AQP8 promotes human dermal fibroblasts to counteract H_2_O_2_-induced oxidative damage. The cells were transfected with siRNA of AQP8 and then exposed to increasing concentrations of H_2_O_2_ (100, 200, 400, 800, and 1,200 μM) for 3 h. (a and b) The effectiveness of siRNA in silencing AQP8 gene expression was validated by Western blot analysis. (c) Intracellular ROS generation was detected using CellROX^TM^ Green Reagent. (d) The gene expression of target gene was identified by qPCR. *p  <  0.05, **p  <  0.01, ***p  <  0.001, as compared to the control. All experiments were performed independently at least three times. Abbreviations: AQP8, aquaporin-8; COL1A1, collagen type I1; KRT19, keratin 19.

As shown in Figures 3 and 4, H_2_O_2_ led to a significant increase in AQP8 gene expression and protein expression in the human dermal fibroblasts CG1639. Transfection with AQP8 siRNA significantly reduced the endogenous protein levels of AQP8 in the cells transfected with AQP8-siRNA (silenced) compared to control cells (Figure 5a and b), confirming the effectiveness of the siRNA approach in silencing the AQP8 gene. Next, the deleterious effects of H_2_O_2_ on the cells transfected with AQP8-siRNA were studied. As shown in Figure 5c, AQP8-silenced cells had higher ROS levels than control cells after H_2_O_2_ treatment. Moreover, AQP8 knockdown led to significantly lower expression of COL1A1, KRT19, and Aggrecan in H_2_O_2_-treated cells (Figure 5d), indicating that AQP8, which plays an important role in H_2_O_2_ transmembrane transport, is upregulated in stressed human dermal fibroblasts to help the cells counteract the H_2_O_2_-induced oxidative damage.

## 4 Discussion

The skin is a highly susceptible target of oxidative stress as a result of ROS generated by both external and internal sources [1]. The effects of aging on the skin are most evident in the dermis layer, where dermal fibroblasts are the primary cell type [25,26]. Previous studies have demonstrated that human dermal fibroblasts are sensitive to oxidative stress-induced cytotoxicity and that H_2_O_2_ exposure leads to a dose-dependent reduction in cell viability, as well as increased ROS levels, indicating that skin cells are facing a significant oxidative challenge [5,23,27]. In addition, oxidative stress has been linked to skin inflammation, and previous research has suggested that aging-associated inflammation may be the result of a decrease in the levels of anti-inflammatory cytokines such as IL-10 [28]. Consistent with this, our study showed that H_2_O_2_ exposure reduced the gene expression of IL-10 in human dermal fibroblasts, which could exacerbate the inflammatory response during oxidative stress, further promoting the aging process.

Recent studies have highlighted the role of pattern recognition receptors, specifically the FPRs, in modulating the sustaining and resolving of inflammatory responses [29]. FPR2, in particular, is an important target for the regulation of the inflammatory response and has been shown to exhibit a preference for ligand-biased signaling during prolonged inflammatory responses [30]. Although FPR2 is primarily expressed in innate immune cells, it has also been observed in non-immune cells such as synovial fibroblasts, keratinocytes, microvascular endothelial cells, and intestinal epithelial cells [29]. Our findings indicate that low-dose H_2_O_2_ treatment upregulates FPR2 expression in human dermal fibroblasts, potentially promoting pro-inflammatory effects. Conversely, high doses of H_2_O_2_ downregulate FPR2, potentially suppressing the resolution of inflammation and preventing inflamed cells from returning to homeostasis. However, further research is necessary to fully elucidate the relationship between H_2_O_2_ exposure and FPR2 activity.

The assessment of COL1A1 and KRT19 as markers of ECM structural integrity is grounded in the understanding of skin aging and the critical role of these proteins in maintaining skin health. For example, COL1A1 is one of the predominant proteins in the dermal ECM, and it serves as a major contributor to tensile strength and structural support [2,31,32]. The degradation of COL1A1 is a hallmark feature of skin aging, leading to decreased skin firmness and the formation of wrinkles and fine lines. Indeed, our results also demonstrated that decreased COL1A1 gene and protein expression correlated with the increased level of H_2_O_2_. KRT19 is an intermediate filament protein that plays a vital role in maintaining the structural integrity of various epithelial tissues, including skin. In our study, COL1A1 and KRT19 were selected as markers because they are significantly affected by H_2_O_2_-induced damage, which mimics oxidative stress, a key contributor to skin aging [33–37]. This damage reduces these essential proteins, contributing to the deterioration of ECM structural integrity. Notably, previous research has indicated that increased expression or supplementation of COL1A1 and KRT19 can promote accelerated wound healing and, by extension, may aid in maintaining or restoring the structural integrity of the ECM [33–37]. This suggests that targeting these markers could have therapeutic implications in mitigating the effects of ECM degradation in the context of skin aging.

Our study found that exposure to H_2_O_2_ dose-dependently decreases COL1A1 expression levels in human dermal fibroblasts. Proteoglycans are also essential for ECM development, organization, hydration, and function [38]. We observed a similar dose-dependent reduction in Aggrecan proteoglycan gene expression following H_2_O_2_ treatment. Additionally, cytoskeletal proteins have been implicated in aging processes [39]. Our results demonstrate that H_2_O_2_ treatment also dose-dependently downregulates the expression of KRT19, a type I epidermal keratin predominant in simple epithelia [40]. These findings are consistent with previous studies [5,23,27] indicating that H_2_O_2-_induced oxidative stress can induce premature senescence and aging in human dermal fibroblasts. As the pro-aging effects of H_2_O_2_ are dose-dependent, regulating H_2_O_2_ permeability in skin cells may be a promising approach for mitigating H_2_O_2_-induced oxidative damage and preventing skin aging. Of note, dermal fibroblasts have two subpopulations, papillary and reticular, which have distinct gene expression profiles (i.e., MMPs, COL1A1, KRT19, and ACAN responsible for cytoskeleton structure and cell mobility. From previous studies and the STR profile of CG1639 cells in this study, it has been shown that this cell type exhibited both a papillary and reticular phenotype [36,41–44]. Therefore, we would like to emphasize that current research focuses on the broader effects of AQP8 on dermal fibroblasts in general, rather than specifically on a single subpopulation.

AQPs are a group of membrane channel proteins that aid in the movement of water and small neutral solutes across biological membranes, and they are gaining recognition as important players in skin physiology and skin disorders. Peroxiporins, which include AQP1, AQP3, AQP5, AQP8, AQP9, and AQP11, are necessary for the efficient transmembrane diffusion of H_2_O_2_, making these channels crucial for H_2_O_2_ performance. Previous research has shown that different skin cell types express various types of AQPs [8,9,45] and that AQP8 provides high efficiency in transporting H_2_O_2_ in HEK-293 cells. Therefore, we specifically focused on AQP8 and provided the first characterization of AQP8 in skin cells, suggesting that exposure to H_2_O_2_ induces the upregulation of AQP8 expression in human dermal fibroblasts, which is indicative of AQP8’s endogenous distribution in the skin.

AQP8 regulates the transport of H_2_O_2_ through the plasma membrane in HeLa cells, particularly during cell stress, suggesting that AQP8 may be a crucial target of stress-induced redox regulatory modification [17,19,46,47]. In this study, knocking down AQP8 leads to a remarkable increase in ROS generation, which in turn significantly decreases the expression of COL1A1, KRT19, and Aggrecan genes. This may be because, without AQP8, the secondary H_2_O_2_ induced by H_2_O_2_ treatment is trapped in the mitochondria, a subcellular site of ROS generation, impeding the efflux of H_2_O_2_ into the cytoplasm. This results in increased H_2_O_2_ levels in the mitochondria, leading to the generation of highly toxic hydroxyl radicals via the Fenton reaction. Although further research is needed to fully understand the biophysical, biochemical, and functional characteristics of AQP8 in skin cells, this study provides a foundation for future work aimed at elucidating the physiological and pathophysiological significance of AQP8 in oxidative stress-induced skin aging and skin diseases.

## 5 Conclusions

In summary, the upregulation of AQP8 expression in response to H_2_O_2_-induced oxidative stress in human dermal fibroblasts can serve as a defense mechanism against the pro-aging effects of oxidative stress. However, when the levels of oxidative stress exceed a certain threshold, the ability of cells expressing AQP8 to eliminate excess H_2_O_2_ may become overwhelmed, resulting in ROS accumulation, oxidative damage, and cell death. Therefore, understanding the regulatory mechanisms that govern AQP8 activity in the skin and identifying compounds that can modulate its function may provide new opportunities for developing preventative and therapeutic strategies for maintaining skin health.

## Supplementary Material

Supplementary material
