# Supplementary material for: Aquaporin-8 promotes human dermal fibroblasts to counteract hydrogen peroxide-induced oxidative damage: A novel target for management of skin aging
Source: Open Life Sci. 2024 Feb 16;19(1):20220828. doi: 10.1515/biol-2022-0828 (PMC10921499; doi:10.1515/biol-2022-0828)
Supplement: Supplementary material [file biol-2022-0828-sm.pdf]

Raw data of Fig.1

Fig. 1B

H2O2 concentration(uM)	Batch#1	Batch#2	Batch#3
0	95	91	90
100	90	82	95
200	85	90	81
400	70	78	72
800	65	70	82
1200	45	51	55

Fig. 1C

Culture Time (h)	Batch#1	Batch#2	Batch#3
0	95	91	90
2	85	89	88
4	81	88	74
8	70	81	75
12	72	61	78
24	34	40	31
48	21	18	15
72	7	9	14

Raw data of Fig.2

H2O2 concentration (uM)	Batch#1	Batch#2	Batch#3	Batch#4	Batch#5	Batch#6
0	0.953538	1.59188	0.680692	0.881188	1.17235	0.844316
100	1.48882	1.2048	1.594824	1.14053	1.3318	2.201154
200	3.13917	2.03755	2.3778	3.95889	1.7806	2.0374
400	2.106385	3.079846	1.474688	2.484667	2.388111	2.435929
800	2.62619	3.036625	2.363364	3.822182	3.008467	2.764941
1200	3.2015	4.1803	3.105154	3.120333	4.125467	3.491563

Raw data of Fig.3

AQP8

0uM	100uM	200uM	400uM	800uM	1200uM
1.00001	0.9619244	3.537336	2.733216	4.990022	6.239669
1.000001	2.556029	4.246643	4.638458	1.9415	3.926098
1	3.877576	3.110136	4.793947	4.855257	4.649189
0.7873477	2.697391	2.964602	1.660503	4.603242	6.663102
1.270087		4.671251		2.948427	

IL-10

0uM	100uM	200uM	400uM	800uM	1200uM
1.0001	0.4553747	0.4206032		0.4601216	0.4059418
1.000001	0.6930205	0.648653	0.7480604	0.6969013	0.6212488
1	0.5902417	0.6098689	0.7829819	0.6662292	0.5731472
0.9394441	0.6175673	0.4911544	0.4103653	0.3088175	0.8514475
0.9496012	0.6503254	0.5382208	0.4650031	0.3161965	0.7471307
1.120954	0.6501542	0.4763101	0.482238	0.2957417	0.9047441

FPR2

0uM	100uM	200uM	400uM	800uM	1200uM
1.00001	1.245204	7.906977	2.65561	0.336891	0.5142726
1.000001	0.6481462	10.11	2.531961	1.400491	0.6560323
1	1.233689	10.07295	1.931606	0.5683617	1.034715
	1.902282	18.11698	2.860132	0.05762826	1.227505
1	2.938652	15.53174	3.677612	0.08128588	1.412222
1.420635	2.788784	14.22094	2.682979	0.209937	1.322469
1.000001	0.6720886	8.346797	4.044882	0.4053149	0.3709379
1	1.272301		4.221992	0.793812	1.372814
1	0.8970502	10.8742	5.440918	1.095204	1.681942

COl1A1

0uM	100uM	200uM	400uM	800uM	1200uM
1.0001	0.5967805	0.4308862	0.5438898	0.6558648	0.4194058
1.00002	0.2446492	0.1238063	0.1696623	0.2131527	0.1616029
1.000003	0.666396	0.3959108	0.4551472	0.5799232	0.4054936
1.17295	0.6964095	0.3603266	0.4027807	0.257524	0.7592198
0.8950052	0.6946223	0.3770352	0.3941544	0.2741266	0.7057785
0.9525657	0.86916	0.3740268	0.4199426	0.3239899	0.619058

KRT19

0uM	100uM	200uM	400uM	800uM	1200uM
1.000001	0.4145239	0.4015279	0.3983384	0.415835	0.3691123
1.0001	0.4660625	0.3830241	0.4201875	0.4159562	0.3826872
1	0.5295961	0.4039689	0.4967571	0.3860729	0.4116887
0.949	0.4090118	0.292	0.2937495	0.1900667	0.5123342
1.087	0.3759619	0.314	0.3068369	0.1873865	0.5578833
0.9701021	0.398	0.2774341	0.3116034	0.173067	0.4814721

Aggrecan

0uM	100uM	200uM	400uM	800uM	1200uM
1.000001	0.39	0.4	0.22	0.29	0.39
1	0.43	0.3312255	0.14	0.15	0.48
1	0.66	0.35	0.16	0.37	0.64
1.0001	0.5923271	0.2307143	0.2950494	0.1679461	0.3782287
1.00002	0.3822248	0.3620252	0.142038	0.4765795	0.2452545
1.000003	1.084451	0.6668124	0.439885	0.6430708	0.5280955

Raw data of Fig.4

H2O2 concentration (uM)	aqp8-1						Col1						KRT					
0	0.637673	0.548482	0.602273	0.725251	0.551664	0.861169	1.025867	1.155976	1.073008	1.166761	1.162682	0.919415	0.826892	0.976162	0.677911	0.940457	0.981826	0.771016
100	0.716446	0.696783	0.721341	0.707731	0.759311	0.740012	1.19406	0.944381	1.187491	1.179535	1.029128	1.095374	1.295159	0.822938	0.724433	1.279404	0.896787	0.668237
200	0.793776	0.79046	0.77012	0.832629	0.832265	0.812532	1.200569	0.660897	1.037866	1.259334	0.69585	0.932176	1.083637	1.030707	1.040663	1.136679	1.085217	0.934689
400	0.731704	0.798397	1.410775	0.797779	0.840327	1.209285	1.052884	0.48221	0.993096	1.147962	0.507535	0.85126	1.154114	0.910108	0.931178	1.258334	0.957905	0.798186
800	0.726298	0.7614	1.052317	0.797631	0.809308	0.882752	0.980245	0.335318	0.665513	1.07652	0.356417	0.558275	0.955327	0.67062	1.646475	1.049154	0.712816	1.38117
1200	0.842981	0.812367	0.775865	0.877189	0.751835	0.831191	0.898805	0.223253	0.478666	0.935279	0.241487	0.420148	0.587119	0.795181	1.176979	0.610945	0.860124	1.033092

Raw data of Fig.5

Fig. 5A

	- siRNA	+ siRNA
Batch1	78	27
Batch2	85	21
Batch3	96	34

Fig. 5C

H2O2 concentration (uM)	Batch1	Batch2	Batch3	Batch4	Batch5	Batch6
-siRNA						
0	0.953538	1.59188	0.680692	0.881188	1.17235	0.844316
100	1.48882	1.2048	1.594824	1.14053	1.3318	2.201154
200	3.13917	2.03755	2.3778	3.95889	1.7806	2.0374
400	2.106385	3.079846	1.474688	2.484667	2.388111	2.435929
800	2.62619	3.036625	2.363364	3.822182	3.008467	2.764941
1200	3.2015	4.1803	3.105154	3.120333	4.125467	3.491563
+siRNA						
0	0.653538	1.29188	0.680692	0.881188	1.17235	0.844316
100	2.8882	2.2048	1.94824	1.4053	2.3318	2.201154
200	3.917	2.755	3.778	3.95889	2.806	2.0374
400	3.106385	3.9846	2.74688	2.84667	3.88111	3.929
800	3.62619	3.6625	3.63364	3.822182	3.8467	2.941
1200	5.2015	4.803	4.5154	3.9333	4.67	3.91563

Fig. 5D

Col1A1

H2O2 concentration (uM)	0uM	100uM	200uM	400uM	800uM	1200uM
Batch1	1.0001	0.5967805	0.1308862	0.0438898	0.0558648	0.04194058
Batch2	1.00002	0.2446492	0.1238063	0.1696623	0.1131527	0.01616029
Batch3	1.000003	0.666396	0.1959108	0.1551472	0.05799232	0.04054936
Batch4	1.17295	0.6964095	0.1603266	0.2027807	0.0257524	0.07592198
Batch5	0.8950052	0.6946223	0.2770352	0.1941544	0.12741266	0.07057785
Batch6	0.9525657	0.86916	0.1740268	0.1199426	0.3239899	0.0619058

KRT19

H2O2 concentration (uM)	0uM	100uM	200uM	400uM	800uM	1200uM
Batch1	1.000001	0.14145239	0.24015279	0.13983384	0.1415835	0.03691123
Batch2	1.0001	0.14660625	0.23830241	0.24201875	0.04159562	0.03826872
Batch3	1	0.15295961	0.24039689	0.14967571	0.03860729	0.0116887
Batch4	0.949	0.14090118	0.292	0.12937495	0.1900667	0.05123342
Batch5	1.087	0.13759619	0.314	0.23068369	0.01873865	0.05578833
Batch6	0.9701021	0.1398	0.12774341	0.3116034	0.173067	0.04814721

Aggrecan

H2O2 concentration (uM)	0uM	100uM	200uM	400uM	800uM	1200uM
Batch1	1.000001	0.239	0.24	0.122	0.029	0.039
Batch2	1	0.143	0.13312255	0.14	0.15	0.048
Batch3	1	0.266	0.135	0.16	0.037	0.064
Batch4	1.0001	0.15923271	0.2307143	0.02950494	0.1679461	0.03782287
Batch5	1.00002	0.3822248	0.13620252	0.142038	0.04765795	0.02452545
Batch6	1.000003	0.84451	0.6668124	0.0439885	0.16430708	0.05280955

Western blot images

ACTIN

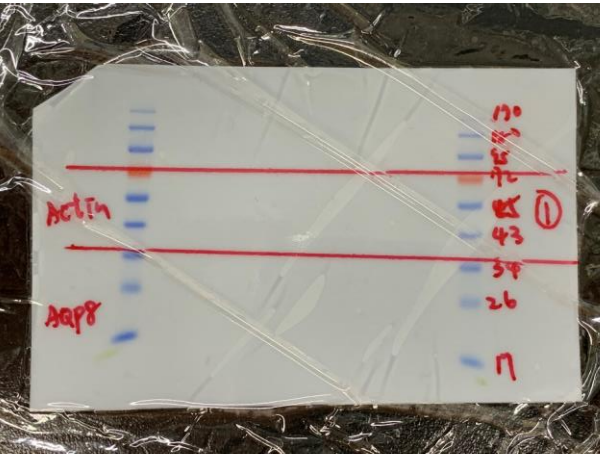

Actin
AQP8

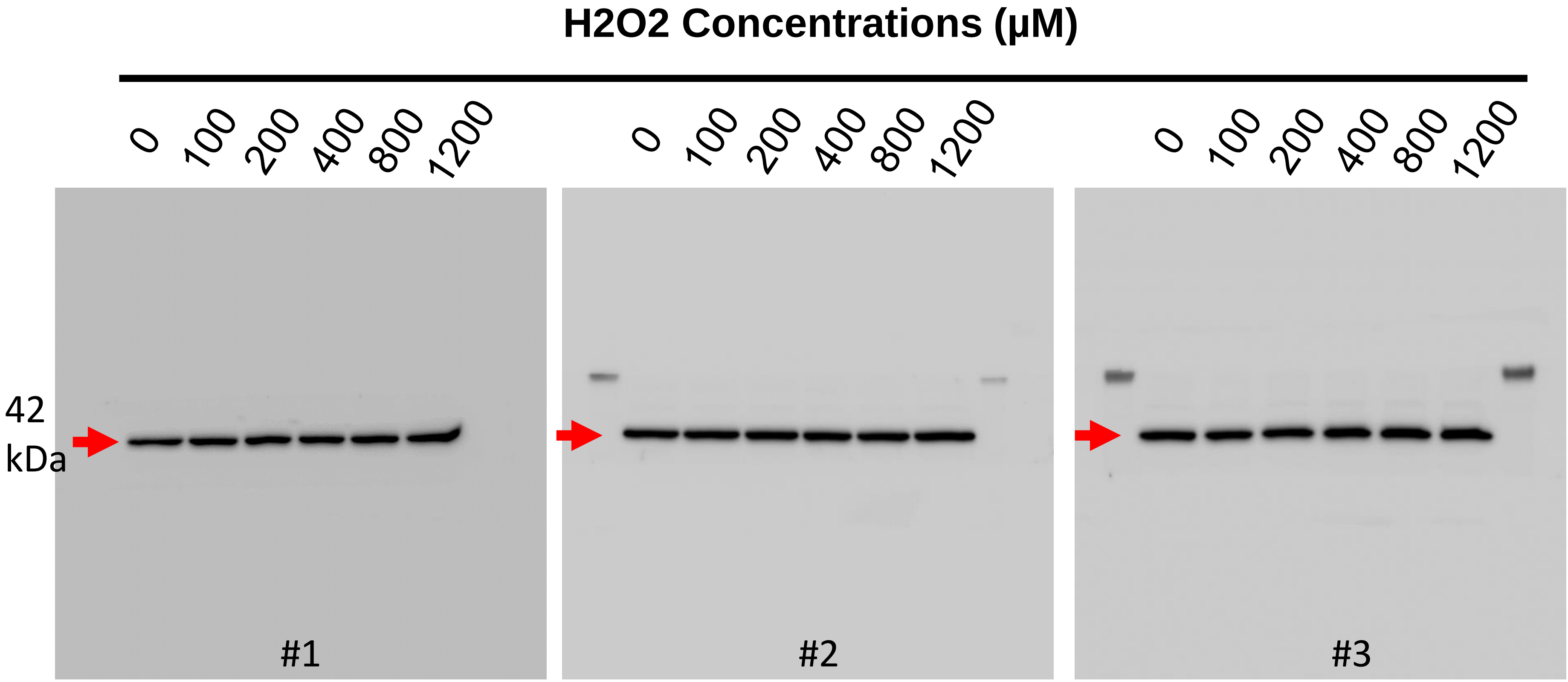

H2O2 Concentrations (μM)
0
100
200
400
800
1200
42 kDa
#1
#2
#3

AQP8

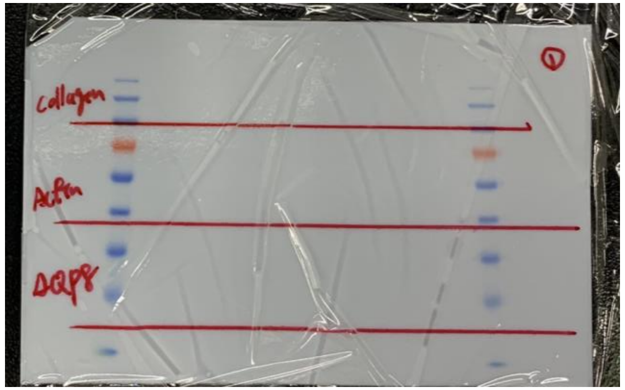
collagen
AQP8

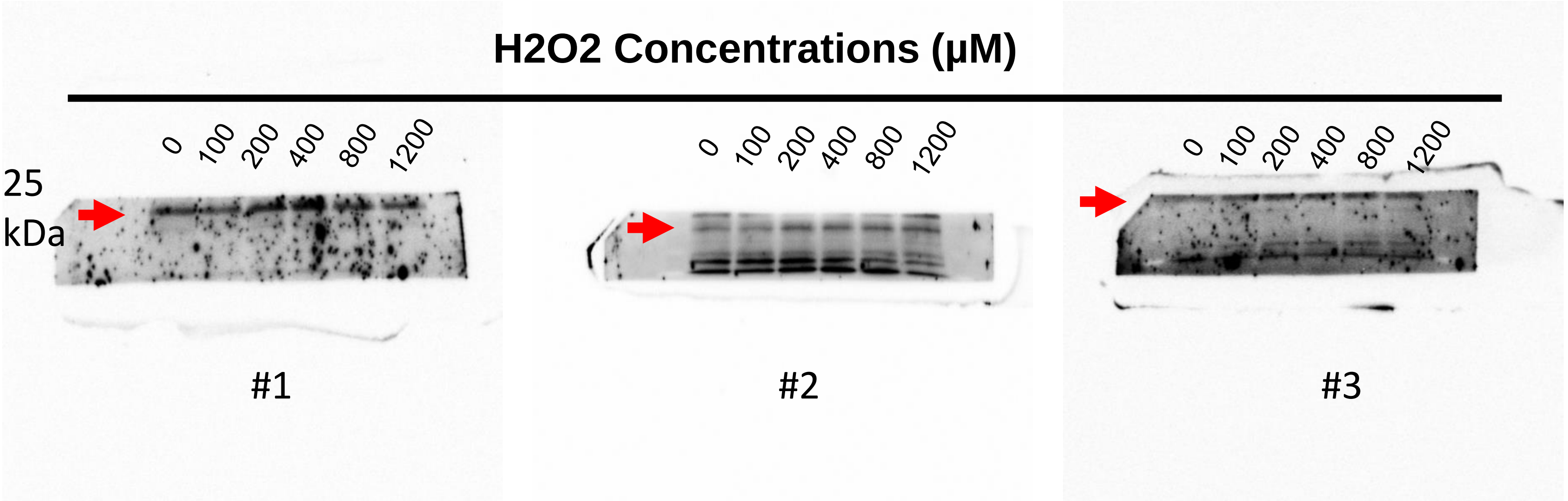
H2O2 Concentrations (µM)
0
100
200
400
800
1200
25
kDa
#1
#2
#3

COL1A1

H2O2 Concentrations (μM)

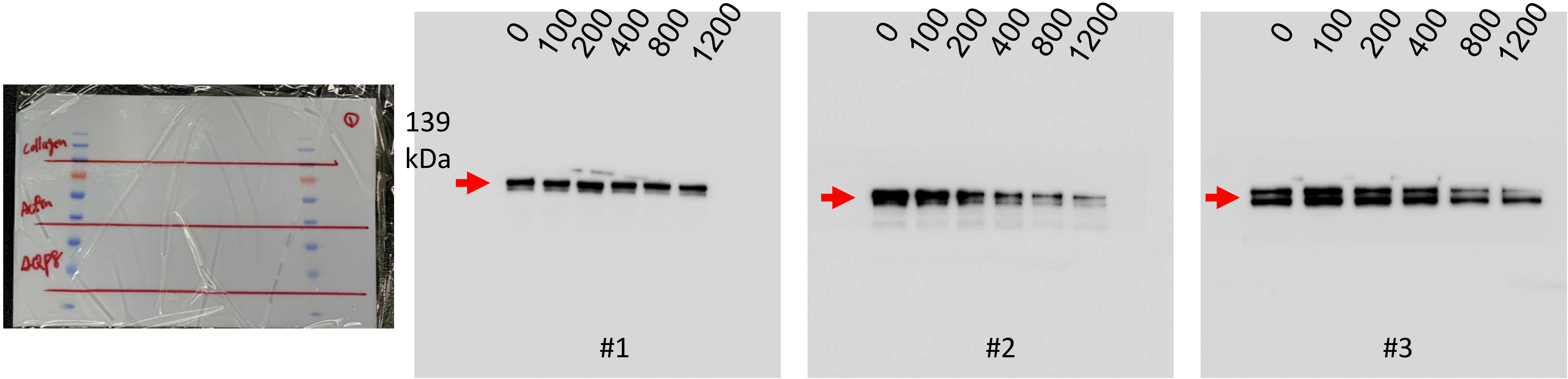

KRT19

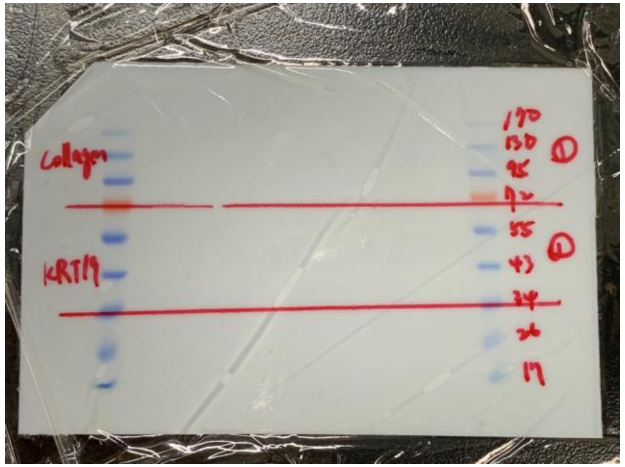
Collagen
KRT19
170
130
95
72
55
43
34
26
17

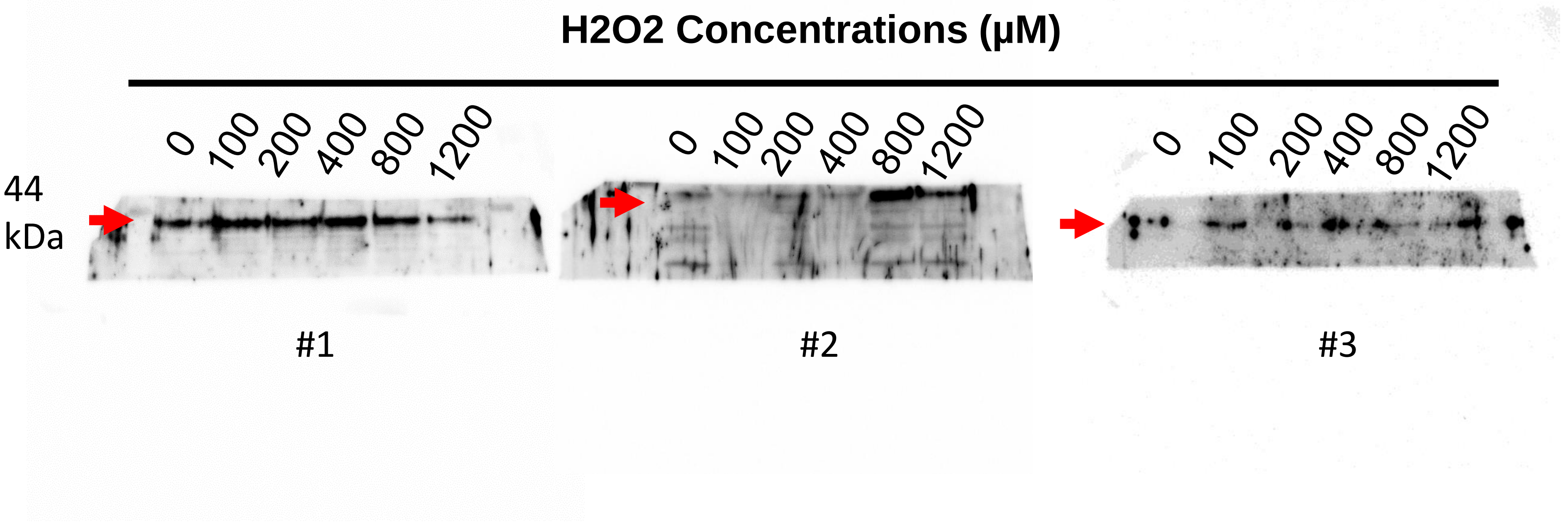
H2O2 Concentrations (µM)
0
100
200
400
800
1200
44
kDa
#1
#2
#3

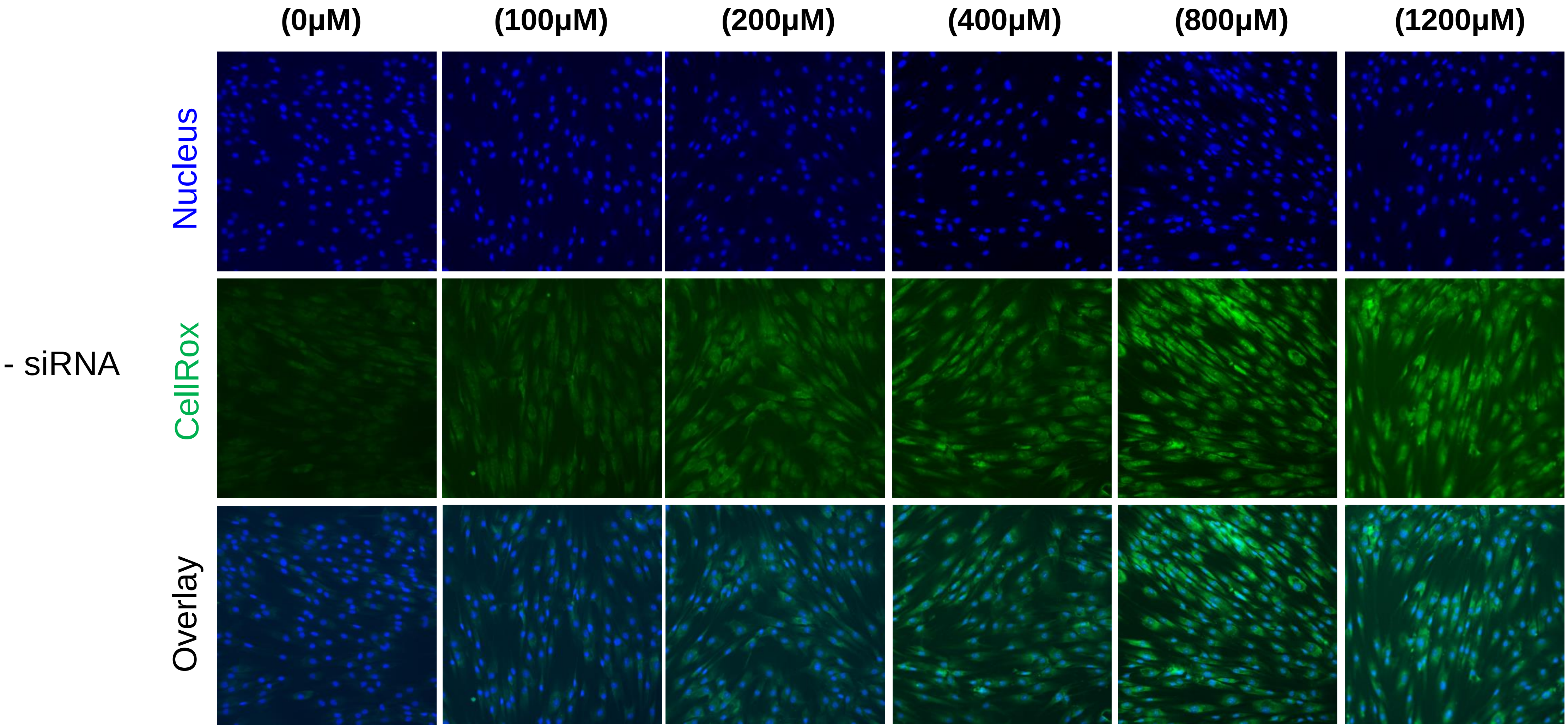
(0μM)
(100μM)
(200μM)
(400μM)
(800μM)
(1200μM)
Nucleus
CellRox
Overlay
- siRNA

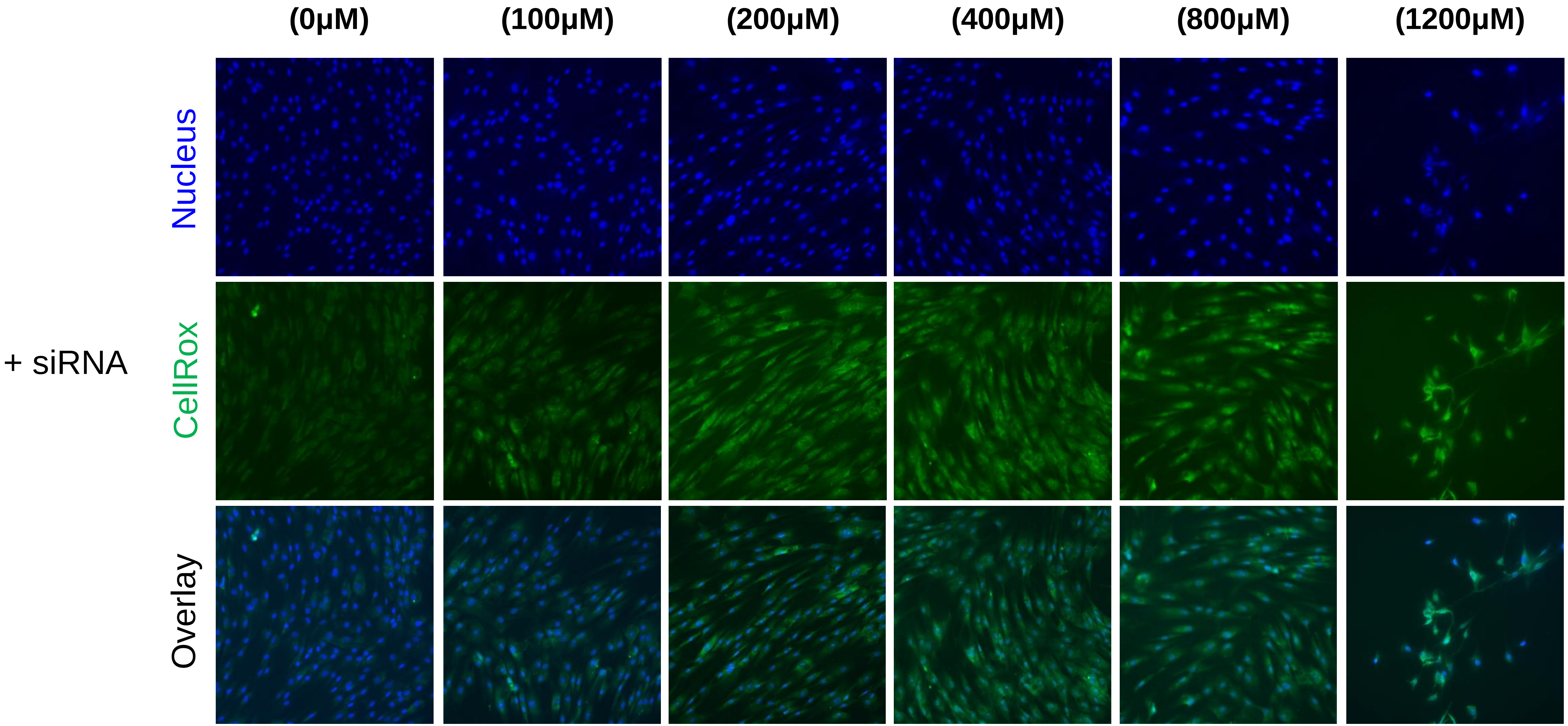
(0μM)
(100μM)
(200μM)
(400μM)
(800μM)
(1200μM)
+ siRNA
Nucleus
CellRox
Overlay
